# Asthma Outcome Measures Before and After the COVID-19 Outbreak Among the Pediatric Population in a Community Hospital

**DOI:** 10.7759/cureus.25621

**Published:** 2022-06-03

**Authors:** Mahrukh Shah, Mohammed Alsabri, Farouk Al-Qadasi, Saadia Malik, Christopher McClean, Khalid Ahmad, Carolyn Springer, Kusum Viswanathan, Fernanda E Kupferman

**Affiliations:** 1 Pediatrics, Brookdale University Hospital Medical Center, Brooklyn, USA; 2 Health and Public Health, Yemen Family Care Association (YFCA), Sanaa, YEM; 3 Pediatrics, Saba University School of Medicine, The Bottom, BES; 4 Pulmonology, Brookdale University Hospital Medical Center, Brooklyn, USA; 5 Psychology, Adelphi University, New York, USA

**Keywords:** patient outcome assessment, african american, pediatrics, asthma, covid-19 pandemic

## Abstract

Background

The severe acute respiratory syndrome coronavirus 2 (SARS-CoV-2) pandemic is a global health threat that has affected patient care enormously. Moderate to severe asthma was listed as a risk factor for severe SARS-CoV-2 disease by the Centers for Disease Control. Little is known about the impact of the pandemic on asthma control in children, particularly African American children.

Objective

The present study sought to determine how changes during the coronavirus disease 2019 (COVID-19) pandemic affected asthma metrics in a majority African American pediatric population at a pediatric pulmonology clinic in a community hospital in New York.

Methods

This is a retrospective, pre-post, comparative cross-sectional study that included children three to 18 years of age with a known diagnosis of asthma followed in a pulmonary clinic. Data were gathered from electronic medical records. Subjects were selected if they presented to a pulmonology clinic within a certain time window both before and after the outbreak of the COVID-19 pandemic. Outcome variables included asthma medication statistics and healthcare utilization statistics.

Results

Inclusion criteria were met by 104 pediatric patients. The majority were African American. Emergency department visits, primary physician visits, and hospitalizations significantly decreased in the post-COVID study group compared to the pre-COVID control group.

Conclusion

Among a majority African American pediatric population, there were significant improvements in asthma outcomes after COVID-19 societal changes when compared to before COVID-19 based on outcome variables.

## Introduction

Asthma is the most common, long-term condition in children, with prevalence rates of 7.5% in the United States among children less than 18 years old in 2018 according to the Centers for Disease Control and Prevention, with the highest prevalence rates among children between 12 and 17 years of age with a slight predominance in boys over girls (8.3% vs. 6.7%, respectively ) [1-2]. The prevalence of childhood asthma with its related burden has been increasing over recent decades worldwide [2-3]. According to national health care use data in 2016, the rate of health care utilization for asthma attacks was 332 physician office visits, 74.3 emergency department (ED) visits, and 10.7 hospitalizations per 10,000 population [2-3]. Frequent asthma attacks in children have been associated with poor quality of life and accelerated loss of lung function [4].

In particular, the morbidity and mortality of pediatric asthma are more prevalent among African American children as compared to Caucasian children and other minorities [5]. The increased rates of asthma among the African American population are due to a multitude of factors, including, but not limited to, socioeconomic factors, obesity, increased exposure to air pollution in inner-city neighborhoods, and even genetics. Minority groups, primarily African American children, have been routinely underrepresented in pediatric asthma research and literature. In late 2019, the novel severe acute respiratory syndrome coronavirus 2 (SARS-CoV-2; coronavirus disease 2019 (COVID-19)), previously called 2019-nCoV, was initially reported in Wuhan, China, and marked the beginning of the current pandemic [6-7]. The Centers for Disease Control and Prevention (CDC) listed moderate to severe asthma as a risk factor for COVID-19 given that asthma in children is commonly triggered by respiratory viruses [8]. However, recent research on the interplay of COVID-19 and pediatric asthma concludes the contrary [8-10]. A research study conducted by Bandi et al. (2020) found no significant difference in the rate of asthma between COVID-positive and COVID-negative cases in pediatric patients [11]. Kenyon et al (2020) reported a 76% reduction in ED visits during the peak of COVID in March-April 2020 as compared to pre-COVID-19 [12].

Similarly, Taquechel et al. (2020) reported that outpatient asthma encounters decreased by 87%, hospital visits decreased by 84%, virtual visits increased greatly, and there was also a significant reduction in asthma-related steroid prescriptions during the pandemic [13]. Given these significant improvements in asthma outcome measures in the general pediatric population, the present study sought to investigate whether these improvements extend to the pediatric population in New York, specifically among a pediatric population with a large percentage of African American children. If significant improvements are maintained in this population, determining the specific factors that contribute to such marked improvement could lead to breakthroughs in improving morbidity and mortality among African American pediatric patients with asthma, a historically overlooked population. The present study also sought to investigate the utilization of telemedicine among this group, which could provide additional tools for providing healthcare to an underserved population.

## Materials and methods

Study design

This retrospective, pre-post, comparative cross-sectional study was conducted at the end of 2020 at a pediatric pulmonary clinic of a community and teaching hospital. The research project was approved by the Institutional Review Board committee of One Brooklyn Heath, Brookdale Hospital Medical Center: Protocol 20-49.

Participants

Children aged three to 18 years old with a diagnosis of asthma who presented to one pulmonology clinic during the time period from March to August 2019 and presented during the time period from March to August 2020 were eligible for this study. Data from 2019 served as the pre-COVID control group while data from the same patients presenting in 2020 served as the post-COVID study group. Children were excluded from this study if they had a history of any significant comorbid disease, such as neonatal chronic lung disease, cystic fibrosis, congenital cardiopulmonary diseases, chronic encephalopathy, or immune deficiency syndrome, along with any disease in which systemic steroid use might be prevalent, including rheumatic disorders, cancer, autoimmune disorders, or cancer.

Data collection

The data were collected from electronic medical records - Epic Software (Epic Systems, Madison, Wisconsin), and data were saved in an Excel sheet (Microsoft Corporation, Redmond, WA). Data from outside of the two selected study time periods from March to August 2019 and 2020 were excluded.

Intervention

The changes that occurred as a result of the COVID-19 pandemic served as an uncontrolled intervention. The researchers in this study served no role in implementing these interventions.

Independent variables

The independent variables were as follows: demographic and socioeconomic factors (patient’s gender, age and race, and asthma category, using biologic agents. In addition, the following variables were included: history of sleeping disorder, food allergy, allergic rhinitis, or eczema.

Outcome variables

The dependent variables in this study were rescue albuterol and systemic steroid use since the previous visit, primary medical doctor (PMD) and emergency department (ED) visits, hospitalizations for asthma exacerbation, pulmonology visits, including telemedicine and in-person visits, change in controller therapy, appropriate spacer use, and compliance with asthma medications. Variables were collected from medical record documentation. Rescue albuterol use and compliance with asthma medications were obtained from patient or parent self-reports along with a medical record review. Rescue albuterol use was considered positive if patients self-reported the need to use rescue albuterol during the study period or if medical records showed any rescue albuterol use during the study period. Compliance with asthma medications was obtained from self-report from patients and was considered positive if the patient or parent-reported regular compliance with medications as prescribed or negative if they reported intermittent compliance or non-compliance. Appropriate spacer use was determined by patient or parent self-report of spacer use at home since the previous visit along with a demonstration of appropriate spacer use to a qualified nurse specialist in person or via a telemedicine visit.

Data management

Data were obtained and compiled from electronic medical records and input into Microsoft Excel. Data were stored only on password-protected hospital computers and de-identified prior to the data analyses, abstracts, and publications. All of the data were confidential and inaccessible to personnel other than study team members.

Adverse events

This study does not involve controlled therapeutic interventions and, therefore, there is no risk involved.

Statistical analysis

This study used Statistical Package for Social Sciences (SPSS) software, version 26 (IBM Corp., Armonk, NY) for statistical analyses. Frequencies and percentages were used to describe the study's qualitative variables and mean and standard deviation were used to present quantitative variables. To assess the association of asthma outcome variables pre and post the COVID-19 pandemic, a paired t-test was conducted for continuous variables and McNemar’s test was used to assess the association in categorical variables. A two-tailed p-value of <0.05 was considered statistically significant.

## Results

A total of 104 asthmatic children aged three to 18 were included in this study. The baseline characteristics of the participants are summarized in Table 1. The mean baseline age of the participants was 9.7±3.8 years, with a slight majority of females over males (52.9% vs. 47.1%, respectively). A majority of the participants, 80 (76.9%), were African American while eight were Hispanic, three were Asian, three were White, and 10 identified as Other. A history of food allergies was present in 36 participants (34.6%). Table 1 baseline characteristics of the study patients (n=104). This table provides the baseline characteristics of the 104 study participants, including the mean age of participants in years, sex, race, and history of food allergies.

**Table 1:** Baseline characteristics of study patients (n=104)

Variable		Mean (SD)	N	%
Age (years)		9.7 (3.8)		
Sex				
	Female		55	52.9
	Male		49	47.1
Race				
	African American		80	76.9
	Hispanic		8	7.7
	Asian		3	2.9
	White		3	2.9
	Others		10	9.6
Food allergies				
	Yes		36	34.6
	No		68	65.4

Table 2 reports several variables associated with asthma, including the category of asthma and any history of allergic rhinitis, eczema, obstructive sleep apnea, biologic agent use, or psychiatric diagnoses. Table 2 also provides p-values for a comparison of these variables between the pre-COVID control group and the post-COVID study group. Participants with active allergic rhinitis significantly decreased from 98 (94.2%) in 2019 to 85 (81.7%) in 2020 (p = 0.001). Participants with active eczema significantly decreased from 28 (26.9%) in 2019 to 19 (18.3%) in 2020. There was no significant increase in the use of biologic agents for asthma in participants, with two using them in 2019 and three using them in 2020. There was no significant difference between the groups for the variables use of biologic agents, obstructive sleep apnea, psychiatric disorders, or categories of asthma.

**Table 2:** Comparison of disease variables pre and post the SARS-CoV-2 pandemic SARS-CoV-2 = severe acute respiratory syndrome coronavirus 2

Variable		Pre-COVID-19		Post-COVID-19		p-value
		N	%	N	%	
Category of asthma						0.114
	Intermittent	10	9.6	5	4.8	
	Mild persistent	27	26	26	25.0	
	Moderate persistent	64	61.5	67	64.4	
	Severe persistent	3	2.9	6	5.8	
Allergic rhinitis						0.001
	Yes	98	94.2	85	81.7	
	No	6	5.8	19	18.3	
Eczema						0.049
	Yes	28	26.9	19	18.3	
	No	76	73.1	85	81.7	
Obstructive sleep apnea						0.064
	Yes	49	47.1	39	37.5	
	No	55	52.9	65	62.5	
Asthmatic biologic agent use						1.000
	Yes	2	1.9	3	2.9	
	No	102	98.1	101	97.1	
Psychiatric disorder						0.375
	Yes	12	11.5	15	14.4	
	No	92	88.5	89	85.6	

There was a significant decrease from the 2019 control group to the 2020 study group in PMD visits for acute asthma exacerbation (mean 0.13 in 2019 to 0.02 visits per study period in 2020, 85% decrease in mean value from 2019 to 2020, p-value 0.01), ED visits for acute asthma exacerbation (mean 0.51 in 2019 to 0.12 visits per study period in 2020, 76% decrease in mean value from 2019 to 2020, p-value <0.001), systemic steroid use for acute asthma exacerbation (mean 0.87 in 2019 to 0.23 steroid courses per study period in 2020, 76% decrease in mean value from 2019 to 2020, p-value <0.001), PICU admissions for asthma (mean 0.08 in 2019 to 0.01 admissions per study period in 2020, p-value 0.034), and hospitalizations (mean 0.13 in 2019 to 0.03 hospitalizations per study period in 2020, 77% decrease in mean value from 2019 to 2020, p-value 0.011), as shown in Table 3. There was no significant change in the overall number of pulmonary visits in each study period. There were no telemedicine pulmonology visits, or 0% of all pulmonology visits, during the study period in 2019 while the mean number of telemedicine visits during the study period in 2020 was 2.29 (89% of all pulmonology visits). There was no significant change in BMI by age-adjusted percentile rank or the overall number of pulmonary visits between the groups.

**Table 3:** Comparison of asthma outcome measures pre- and post-CoV-Sars-2 pandemic Variables are provided as an average number among all participants during a given study period in units of events per study. The variable “all pulmonary visits” includes telemedicine visits and in-person clinic visits. PMD = primary medical doctor; ED = emergency department; PICU = pediatric intensive care unit; BMI = body mass index

Variable	Pre-COVID-19		Post-COVID-19		P-value
	Mean	SD	Mean	SD	
PMD visits for acute asthma exacerbation	0.13	0.34	0.02	0.14	0.010
ED visits for acute asthma exacerbation	0.51	0.90	0.12	0.43	<0.001
Systemic steroid use for acute asthma	0.87	1.01	0.23	0.61	<0.001
Pulmonary telemedicine visits	0.00	0.00	2.29	1.35	-
All pulmonary visits	2.58	1.66	2.57	1.76	0.719
PICU admissions for asthma	0.08	0.30	0.01	0.10	0.034
BMI by percentile rank	70.12	29.41	73.31	29.99	0.091
Hospitalizations	0.13	0.37	0.03	0.22	0.011

The number of participants who had any rescue albuterol use decreased significantly from 102 (98.1%) in 2019 to only 44 (42.3%) in 2020 (p-value <0.001), as seen in Table 4. The number of participants using a spacer at home with a demonstration of the appropriate technique significantly increased from 82 (78.8%) in 2019 to 95 (91.3%) in 2020, representing a 12.5% increase. There was a significant difference in changes in controller therapy among the two groups (p-value 0.03), with 57 participants stepping up medication and six participants stepping down medication in 2019 compared to 16 participants stepping up and 31 participants stepping down medication in 2020. There was no significant change in compliance with asthma medication between groups (65.3% in 2019 vs. 72.7% in 2020, p-value 0.33).

**Table 4:** Comparison of asthma medication usage and technique pre and post-SARS-CoV-2 pandemic SARS-CoV-2 = severe acute respiratory syndrome coronavirus 2

Variable		Pre-COVID-19		Post-COVID-19		p-value
		N	%	N	%	
Rescue albuterol use						<0.001
	Yes	102	98.1	44	42.3	
	No	2	1.9	60	57.7	
Compliance with asthma medication						0.33
	Yes	62	65.3	72	72.7	
	No	33	34.7	27	27.3	
Appropriate spacer use						0.001
	Yes	82	78.8	95	91.3	
	No	22	21.2	9	8.7	
Change in control therapy						0.03
	No change	41	39.4	57	54.8	
	Step up	57	54.8	16	15.4	
	Step down	6	5.8	31	29.8	

## Discussion

This retrospective, pre-post, comparative cross-sectional study of 104 majority African American pediatric patients with asthma before and after the changes caused by COVID-19 shows that there were significant improvements in asthma outcomes after COVID-19 societal changes when compared to before COVID-19 based on PMD visits, ED visits, hospitalizations, PICU admissions, and courses of systemic steroids for asthma exacerbations. These findings are consistent with similar studies performed on other populations of asthma patients [14]. We also found that, at least during the study period, these improvements in asthma outcomes occurred despite an increased reliance on telemedicine pulmonology visits during the study period in 2020, when 89% of all pulmonology visits were done by telemedicine, as opposed to in-person pulmonology visits during the study period in 2019, when all pulmonology visits were conducted in person. Follow-up studies would be needed to examine the long-term effects of changes in telemedicine reliance; however, the implication is that telemedicine may be capable of providing equal or superior care compared to in-person visits under certain circumstances. The African American pediatric population is well-known for an increased prevalence of asthma, increased morbidity and mortality from asthma, substandard levels of care from varying causes, and a paucity of representation in scientific research and literature when compared to other racial groups [15]. The present research shows that significant improvements in asthma outcomes among African American pediatric patients is an attainable goal and helps point to possible critical areas on which to focus future efforts. The first such area includes all the societal changes resulting from COVID-19. The changes are innumerable and may include increased vigilance with regard to sanitation and hygiene, increased awareness of medical conditions and vulnerability to disease, increased compliance with medications, increased utilization of non-emergency medical resources, avoidance of emergency medical resources, changes in physical exercise, changes in environmental exposures, increased social distancing, and isolation, closure of schools, decreased spread of viral disease, wearing of masks in public, public health campaigns, changes in societal norms, and changes in the levels of and exposure to various allergens. It is beyond the scope of this study to innumerate these changes and investigate which of these changes specifically may have contributed to the improvement in asthma outcomes. It can be said, however, that there were significant improvements in asthma outcomes among the majority African American population in this study during the study period in 2020 compared to 2019, and that the societal changes that occurred as a result of COVID-19 are among the possible variables influencing these improvements. Similar prior research has studied the effect of these changes on other patient populations, one such which showed decreased utilization of the ED among patients with asthma in Rhode Island [16]. Other areas that may have influenced the improvement in asthma outcomes include increased appropriate use of spacers for the delivery of asthma medications. We found that the number of participants that were using spacers at home and demonstrated appropriate use of a spacer in the clinic or via telemedicine visit improved from 78.8% (n = 82) in 2019 to 91.3% (n = 95) in 2020. This represents a 12.5% increase, which may account for some of the improvement in asthma outcome measures but may not account for all of the improvement given that ED visits decreased by 76%, systemic steroid use decreased by 76%, hospitalizations decreased by 77%, and PMD visits for asthma decreased by 85% from 2019 to 2020. Appropriate use of controller medications is a known barrier to proper asthma control among pediatric patients and especially the African American pediatric population in general [15]. This study shows that improvements in appropriate spacer use may be associated with improved asthma outcomes in the short term. On the other hand, we found no significant difference in the number of participants self-reporting compliance with medications in 2019 compared to 2020. Therefore, compliance with asthma medication regimens, in general, is not likely to have influenced the improvements in asthma outcome measures seen in this study.

Another known barrier to adequate patient care among the pediatric population and especially impacting the African American population is missed office visits [17]. Similarly, visits to specialty care providers are more likely to be missed than visits to PMDs. Telemedicine provides one possible avenue of reducing the barriers to regularly attending office visits as scheduled in theory. If telemedicine can provide a similar quality of care as that of an office visit, under the appropriate clinical circumstances, it could prove to be a valuable tool in combating the systemic inequities which adversely impact the African American pediatric population and in reducing the barriers to receiving appropriate medical care. The present study found that there were no telemedicine pulmonology visits among the study population during the study period in 2019 while in 2020, the mean number of telemedicine visits during the study period was 2.29, representing 89% of all pulmonology visits during the study period in 2020. At the same time, there was no significant change in the overall number of pulmonology visits between the two groups. In other words, despite an increased utilization of telemedicine in 2020, there was no decrease in the total number of pulmonology visits in 2020 compared to 2019. This is in contrast to a study on telemedicine in surgery clinics, which showed that the odds ratio for African American patients to attend acute care and general surgery clinics was 0.24 compared to a White reference population, which was significant. If true, telemedicine might increase the health disparity instead of decreasing it [18]. Nevertheless, even if the health disparity between the White population and African American population increases, if the total population of African Americans who are able to obtain access to quality healthcare was to increase as a result of increased telemedicine usage such a change would still be beneficial to the African American community and presumably save lives and improve quality of life.

Similarly, despite the large change to telemedicine in 2020 compared to 2019, asthma outcomes improved among the study population, which shows that telemedicine can be effective in managing asthma in this population in the short term. It also shows that this population is largely willing and able to make the adjustment to telemedicine as opposed to in-person visits, given that the total number of pulmonology visits was not significantly different between the two groups. This provides some initial data on the feasibility and effectiveness of the widespread use of telemedicine in the routine treatment of asthma among a majority African American population.

Strengths and limitations

There are some significant limitations of this study. First, the influence of significant confounding variables cannot be excluded. On the other hand, because this was a pre-post study using the same participants in both arms, one strength of this study is that differences between groups are minimal, including genetic differences. The main exception to this is a difference in age between the groups. Because the control arm was performed a year before the study arm, age cannot be excluded as a significant confounder. The results of this study may not be generalizable to other study populations. Self-reporting from patients and parents can result in recall bias.

Implications

This study shows that significant improvements in asthma outcomes are possible in the African American pediatric population, a population that has historically been underrepresented in improvements in healthcare and research. There are many possible reasons for this, which include improved use of spacers, improved self-awareness of medical conditions, improved hygienic care, changes in exposures to allergens, decreased exposure to viral illnesses, social distancing, and mask-wearing. Determining which variables are responsible for these improvements could help guide future routine care of asthma among this population, reducing the morbidity and mortality of asthma among the African American population as a result.

Future directions

Follow-up studies are needed to investigate whether improvements observed during the COVID-19 era remain in place once society returns toward normalcy or whether asthma outcome measurements return to pre-COVID-19 levels. Such research would help determine whether the societal changes themselves are the primary influencer of improved outcomes or whether other variables are responsible. Similarly, studies on the specific changes that may have influenced asthma outcomes could help guide future patient care, such as increased awareness of medical conditions, increased vigilance with regard to self-care and exposure to risky environments, mask-wearing, changes in levels of exposure to various allergens, changes in the home environment, avoidance of medical care, improved patient education, and improved medication compliance. Finally, follow-up studies on the effects of telemedicine pulmonology care on asthma outcomes compared to in-person visits, barriers to telemedicine usage among the African American population, and studies on the rates of telemedicine usage among African Americans compared to in-person visits, may help provide valuable new tools for clinicians and patients and reduce the barriers to medical care among patients that are impacted by systemic inequities and inequalities.

## Conclusions

This study showed that among a majority African American pediatric population, there were significant improvements in asthma outcomes after COVID-19 societal changes when compared to before COVID-19-based on PMD visits, ED visits, hospitalizations, PICU admissions, and courses of systemic steroids for asthma exacerbations despite an increased reliance on telemedicine pulmonology visits.
